# Rolf Christian Gaillard (1944–2011): A builder of modern European endocrinology and neuroendocrinology

**DOI:** 10.1111/jne.70245

**Published:** 2026-08-11

**Authors:** Philippe Chanson, Eduardo Spinedi, François Pralong

**Affiliations:** ^1^ Service d'Endocrinologie et des Maladies de la Reproduction Université Paris‐Saclay, Inserm, Physiologie et Physiopathologie Endocriniennes, Assistance Publique‐Hôpitaux de Paris, Hôpital Bicêtre, Centre de Référence des Maladies Rares de l'Hypophyse Le Kremlin‐Bicêtre France; ^2^ Centre for Experimental and Applied Endocrinology (CENEXA‐UNLP‐CONICET‐CICPBA) La Plata Medical Faculty, La Plata National University La Plata Argentina; ^3^ Service d'Endocrinologie Diabétologie et Obésité, Hôpital de La Tour Meyrin Switzerland

**Keywords:** Hypothalamo‐pituitary adrenal axis, mentorship, neuroendocrinology, neuroimmunoendocrinology, stress hormones

## Abstract

Rolf Christian Gaillard was a Swiss physician, scientist, and academic leader whose career contributed significantly to the development of modern European endocrinology and neuroendocrinology. Among his most remarkable qualities was an exceptional talent for organisation and institution‐building. Together with his collaborators, Gaillard became internationally recognised for his work on regulation of the hypothalamic–pituitary–adrenal axis. His research demonstrated how immune mediators influence endocrine regulation during infection, inflammation, trauma, and chronic stress. His scientific interests extended beyond neuroendocrinology and included leptin physiology, obesity, appetite regulation, adrenal disorders, adult growth hormone deficiency, metabolic diseases, reproductive endocrinology, and neuroendocrine adaptation. A founding member of European Neuroendocrine Association (ENEA), he subsequently served as its Treasurer and then as its President and largely contributed the success of the ENEA Congresses and Workshops.

## 1 INITIAL YEARS

Rolf Christian Gaillard was a Swiss physician, scientist, and academic leader whose career contributed significantly to the development of modern European endocrinology and neuroendocrinology. Among his most remarkable qualities was an exceptional talent for organisation and institution‐building.

Rolf Christian Gaillard was born on 22 May 1944, in Morat (Canton of Fribourg), Switzerland. He studied medicine at the University of Geneva, where he also completed part of his postgraduate clinical and scientific training. During these formative years, he developed a strong interest in internal medicine and endocrine disorders under the mentorship of Professor Alex Muller (1921–2006), founder of the Department of Internal Medicine at Geneva University Hospital and widely regarded as one of the pioneers of clinical investigation in Switzerland. Under his supervision and that of Michel Vallotton, Rolf C. Gaillard conducted research on the regulation of aldosterone in humans. The findings were published in 1976 in what became his first publication as first author.^1^

## 2 EARLY ACADEMIC YEARS IN GENEVA

Following Professor Muller's advice, Gaillard pursued advanced clinical and research training abroad during his postgraduate years. He spent 2 years as a Clinical Research Fellow at St Bartholomew's Hospital in London, one of Europe's oldest and most prestigious teaching hospitals. Under the supervision of Lesley H. Rees and Michael Besser, he investigated the secretion of ACTH, LPH, and *β*‐endorphin by perfused isolated human pituitary tumour cells in vitro.

It was during this period that he developed a lifelong friendship and scientific collaboration with Ashley Grossman. Together, they co‐authored several influential publications on the opioid regulation of gonadotropins, prolactin, growth hormone (GH), thyroid‐stimulating hormone (TSH), and ACTH secretion. Rolf also contributed to the demonstration that angiotensin II stimulates ACTH release from rat anterior pituitary cells.^2^ These studies were awarded by a Price from the Swiss Society of Internal Medicine in 1982.

His years in London exposed him to some of the most distinguished clinical endocrinologists of the time and to internationally recognised research programmes in endocrinology, metabolism, and physiology. This experience played a pivotal role in shaping both his scientific vision and clinical approach.

He subsequently obtained a research fellowship in the Laboratory of Neuroendocrinology directed by Roger Guillemin at the Salk Institute in La Jolla, California. Guillemin had been awarded the 1977 Nobel Prize in Physiology and Medicine, together with Rosalyn Yalow and Andrew Schally, for their pioneering work on hypothalamic hormones.

Gaillard arrived shortly after the landmark discovery of growth hormone‐releasing hormone (GHRH), isolated from a pancreatic tumour causing acromegaly; this relevant finding was reported in *Science* during 1982.^3^ He brought with him four human hypothalamic specimens from Geneva, which enabled the demonstration that the GHRH isolated from the pancreatic tumour was identical to the endogenous chemical and biological forms present in the human hypothalamus.^4^

However, his stay at the Salk Institute was shorter than anticipated. At the request of Professor Muller, he returned to Switzerland after only 10 months to assume the position of *Chef de Clinique Médicale* at Geneva University Hospital. Simultaneously, he was entrusted with establishing the Laboratory of Experimental Neuroendocrinology. In 1989, he was appointed Adjunct Professor at the University of Geneva.

Already recognised as an outstanding clinician, Gaillard introduced combined pituitary stimulation tests into clinical practice in Switzerland. During this period, his major clinical research focus was RU‐486 (mifepristone), a synthetic 19‐norsteroid possessing both antiprogesterone and antiglucocorticoid properties and now widely used for medical termination of early pregnancy.

Soon after its synthesis, he obtained access to the compound from Roussel‐UCLAF and administered it to healthy male volunteers. He demonstrated a dose‐dependent increase in plasma ACTH, *β*‐endorphin, and cortisol concentrations, reflecting disinhibition of the hypothalamic–pituitary–adrenal (HPA) axis. Interestingly, this effect was observed only during morning hours. He further demonstrated that administration of RU‐486 together with dexamethasone at midnight completely abolished the suppressive effect of dexamethasone on pituitary–adrenal activity. This landmark study, published with Étienne‐Émile Baulieu in the *Proceedings of the National Academy of Sciences of the USA* in 1984,^5^ established that the antiglucocorticoid effects of mifepristone occur at doses higher than those required for its antiprogesterone activity. These findings were instrumental in reassuring clinicians regarding the safety profile of the drug when used for fertility control while simultaneously opening new therapeutic perspectives for the treatment of disorders associated with cortisol excess.

In collaboration with colleagues from the Department of Dermatology in Geneva, Gaillard demonstrated that orally administered RU‐486 antagonised the cutaneous vasoconstrictive effects induced by topical glucocorticoids.^6^

Starting in February 1988, he initiated a highly productive scientific collaboration with Eduardo Spinedi, an Argentinian researcher from CONICET‐La Plata, who joined the Laboratory of Experimental Neuroendocrinology as Assistant Professor from the Geneva Faculty of Medicine and subsequently assumed a leading role within the laboratory.

Together, they investigated the effects of adrenalectomy in rats and demonstrated that the median eminence represents a major site of glucocorticoid feedback regulation of the HPA axis through inhibition of CRH and AVP release.^7^ They subsequently expanded their work to investigate the influence of cytokines released by activated immune cells on HPA‐axis function. With this aim, using rat medial basal hypothalamic explants, containing the median eminence, they examined the effects of several cytokines, including interleukin‐1*β* (IL‐1*β*), interleukin‐6 (IL‐6), tumour necrosis factor‐*α* (TNF‐*α*), thymosin fraction 5, and bacterial lipopolysaccharide (LPS), on CRH and AVP secretion. Their findings demonstrated that CRH neurons, rather than vasopressinergic neurons, play a central role in neuroimmune communication.^8^

Gaillard and Spinedi also showed that HPA‐axis activation induced by LPS varied according to sex in BALB/c mice. Female animals exhibited greater ACTH and corticosterone responses, which were further enhanced following gonadectomy, suggesting a centrally mediated mechanism.^9^

At a time when relatively few investigators were exploring direct interactions between the immune and endocrine systems, Gaillard helped pioneer this emerging interdisciplinary field. Supported by substantial funding from the Swiss National Science Foundation, he established several long‐term research programmes that contributed significantly to the development of neuroimmunoendocrinology.

## 3 LEADERSHIP AT LAUSANNE

**FIGURE 1:**
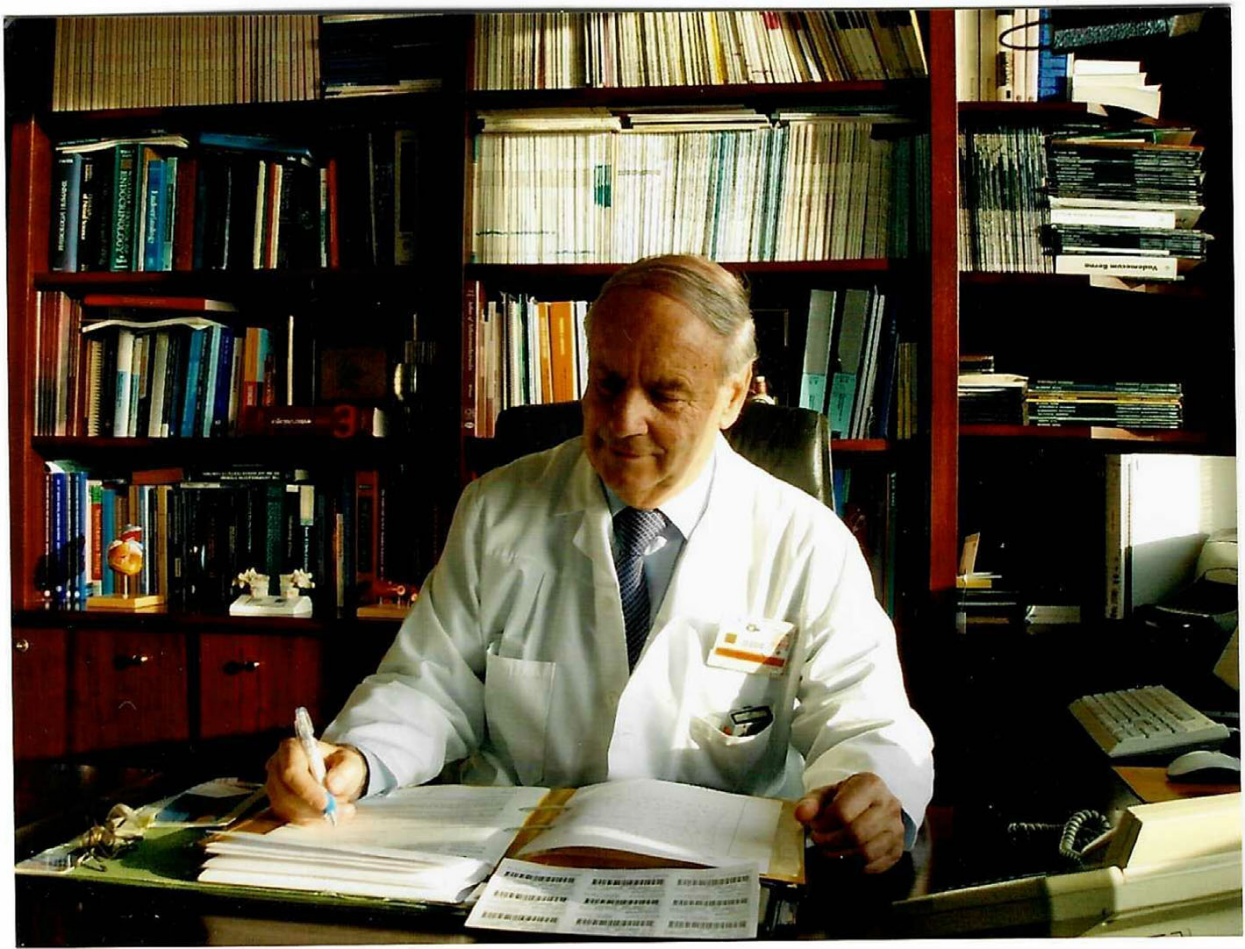
Rolf C. Gaillard in his office at Lausanne University Hospital.

In 1992, Rolf Gaillard was appointed Full Professor at the Faculty of Biology and Medicine of the University of Lausanne and Head of the Department of Endocrinology, Diabetology and Metabolism at Lausanne University Hospital (CHUV), succeeding Professor Thérèse Lemarchand‐Béraud. Under Rolf's leadership, the department became one of Switzerland's leading centres for endocrinology and gained increasing international recognition (Figure 1).

He modernised the department by introducing molecular endocrine research methodologies, developing translational research programmes, expanding research activities in diabetes and obesity, strengthening interactions between laboratory science and clinical medicine, and fostering collaborations with major European medical institutions.

Gaillard firmly believed that medicine should integrate scientific discovery with clinical practice. He encouraged physicians to engage actively in research and promoted multidisciplinary collaborations among endocrinologists, immunologists, neurologists, and molecular biologists. These efforts were supported by several prestigious grants from the Swiss National Science Foundation.

Together with his collaborators, Gaillard became internationally recognised for his work on regulation of the HPA axis. His research demonstrated how immune mediators influence endocrine regulation during infection, inflammation, trauma, and chronic stress.

He continued to work intensely with Eduardo Spinedi, a collaborative programme that lasted for more than 20 years. In this regard, Rolf and Eduardo were the first team demonstrating a direct relationship between the rat HPA axis and the regulation of the white adipose endocrine tissue, mainly in terms of in vivo leptin secretion due to a physiological role of ACTH in a clear regulatory loop.^10^ Rolf and Eduardo pioneered evidence for a neuroendocrine‐immunological sexual dimorphism in rodents.^11^ Additionally, they studied, by using several in vivo models, endotoxic shock‐induced inflammation on metabolic, neuroendocrine and immune functions in undernourished individuals.^12^ Later on, using a rat model of hypothalamic obesity, they were able to demonstrate a role of the chronic excess of white adiposity as a modulator of neuroendocrine‐metabolic‐immune functions under endotoxemia.^13^ By the unfortunate time before Rolf died, they published data emerging from the rat intake of an unbalanced (fructose‐enriched) diet as a detrimental modifier of parametrial adipose tissue functionality, thus establishing overall dysmetabolism.^14^

His scientific interests extended beyond neuroendocrinology and included leptin physiology, obesity, appetite regulation, adrenal disorders, adult growth hormone deficiency, metabolic diseases, and neuroendocrine adaptation.

At Lausanne, much of the scientific activity initiated by Rolf Gaillard was developed in close collaboration with François Pralong, one of his most trusted colleagues and former trainees. Their research encompassed both basic and clinical endocrinology. Experimental studies on hypothalamic neurons provided important insights into the regulation of neuropeptide Y (NPY) by ghrelin, glucocorticoids, and metformin, as well as the control of gonadotropin‐releasing hormone (GnRH) secretion by leptin and NPY.^15^, ^16^ Other investigations focused on the adrenal gland and demonstrated that leptin inhibits glucocorticoid secretion through downregulation of steroidogenic acute regulatory protein (StAR) expression,^17^ thereby suggesting a potential role for leptin in food‐dependent Cushing's syndrome.^18^

In parallel, molecular studies of adipose tissue from obese women revealed differential expression of peroxisome proliferator‐activated receptor gamma (PPAR*γ*) isoforms 1 and 2 and highlighted important molecular differences between visceral and subcutaneous fat depots.^19^, ^20^

François Pralong also established a strong clinical research programme in reproductive endocrinology, contributing to the characterisation of hypogonadotropic hypogonadism through the identification of novel inactivating mutations of the GnRH receptor^21^ and luteinizing hormone *β*‐subunit genes,^22^ as well as through studies of families carrying DAX1 mutations.^23^

Rolf Gaillard fostered a highly productive academic environment that enabled many collaborators to develop their own successful research programmes. Among them, V. Giusti made important contributions to the understanding of obesity and the metabolic consequences of Roux‐en‐Y gastric bypass and gastric banding procedures. J. Ruiz advanced knowledge of diabetes mellitus and its complications, while F. Gomez conducted studies on pituitary apoplexy, the antitumoral effects of cabergoline in large non‐functioning pituitary adenomas, and the diagnosis and outcomes of patients with pituitary tumours and other intrasellar lesions.

Beyond his own scientific achievements, Rolf Gaillard possessed an exceptional clinical acumen. His ability to recognise unusual clinical presentations and establish accurate diagnoses often opened the door to fruitful collaborations with leading international experts. These collaborations resulted in several landmark publications in high‐impact journals. One notable example was his work with C. Stratakis and J. Bertherat on a family affected by micronodular adrenocortical disease and Cushing's syndrome, which led to the identification of mutations in the phosphodiesterase 11A4 (PDE11A) gene.^24^

Rolf Gaillard also played a prominent role in large international post‐marketing studies, particularly within the KIMS programme. Through these activities, he contributed substantially to improving knowledge of long‐term outcomes in adults with growth hormone deficiency receiving GH replacement therapy. Fittingly, his final scientific contribution, published in 2012, 1 year after his death, addressed mortality in GH‐deficient adults treated with growth hormone replacement. In recognition of his major involvement in this work, he was listed as first author, providing a poignant conclusion to a distinguished career devoted to clinical excellence, scientific rigour, and the advancement of endocrinology.^25^

## 4 TEACHER AND MENTOR

Gaillard was known not only as an accomplished scientist but also as a demanding and inspiring teacher. At Lausanne, he trained generations of endocrinologists, internists, and physician‐scientists.

He supervised numerous doctoral theses, postgraduate fellows, clinical trainees, and young investigators. Former students consistently described him as intellectually rigorous, visionary, and deeply committed to academic medicine.

He strongly believed that future medical progress would depend upon interdisciplinary collaboration and translational research, concepts that became increasingly influential throughout the 1990s and 2000s.

## 5 AN ACTIVE REPRESENTATIVE OF SWITZERLAND AND A TIRELESS LEADER OF INTERNATIONAL ORGANISATIONS

As emphasised by Albert Beckers in his obituary published in *Pituitary* in 2012, “Rolf will be best remembered as a builder of the clinical specialty of endocrinology in Europe” and for his major contributions to the development of European endocrine and neuroendocrine organisations.^26^

His numerous leadership positions included:

- President of the Swiss Society of Endocrinology (1987).
- Active member of the Endocrinology Section of the Union of European Medical Specialists (UEMS) from 1994 onward. He served as Secretary‐Treasurer of the European Board of Endocrinology in 1998 and became President of the UEMS Endocrinology Section in 2002.
- Founding member of the European Neuroendocrine Association (ENEA) in 1984, member of its Executive Committee, Treasurer and finally President until his death. As noted by Beckers, “He worked tirelessly on all aspects of the ENEA, including the success of the ENEA Congresses and Workshops. He continued to contribute ideas and inspiration for all activities of the ENEA right up to his final months”.^26^
- Member of the Executive Board of the *Journées Internationales d'Endocrinologie Clinique Henri‐Pierre Klotz*, representing Switzerland, and President of the organisation from 2006 to 2009.
- Member of several international editorial boards and scientific advisory committees, including programmes supported by Novartis and Pfizer (KIMS and ACROSTUDY).
- Elected member of the Executive Committee of the European Society of Endocrinology in 2006.

## 6 DISTINCTIONS

**FIGURE 2:**
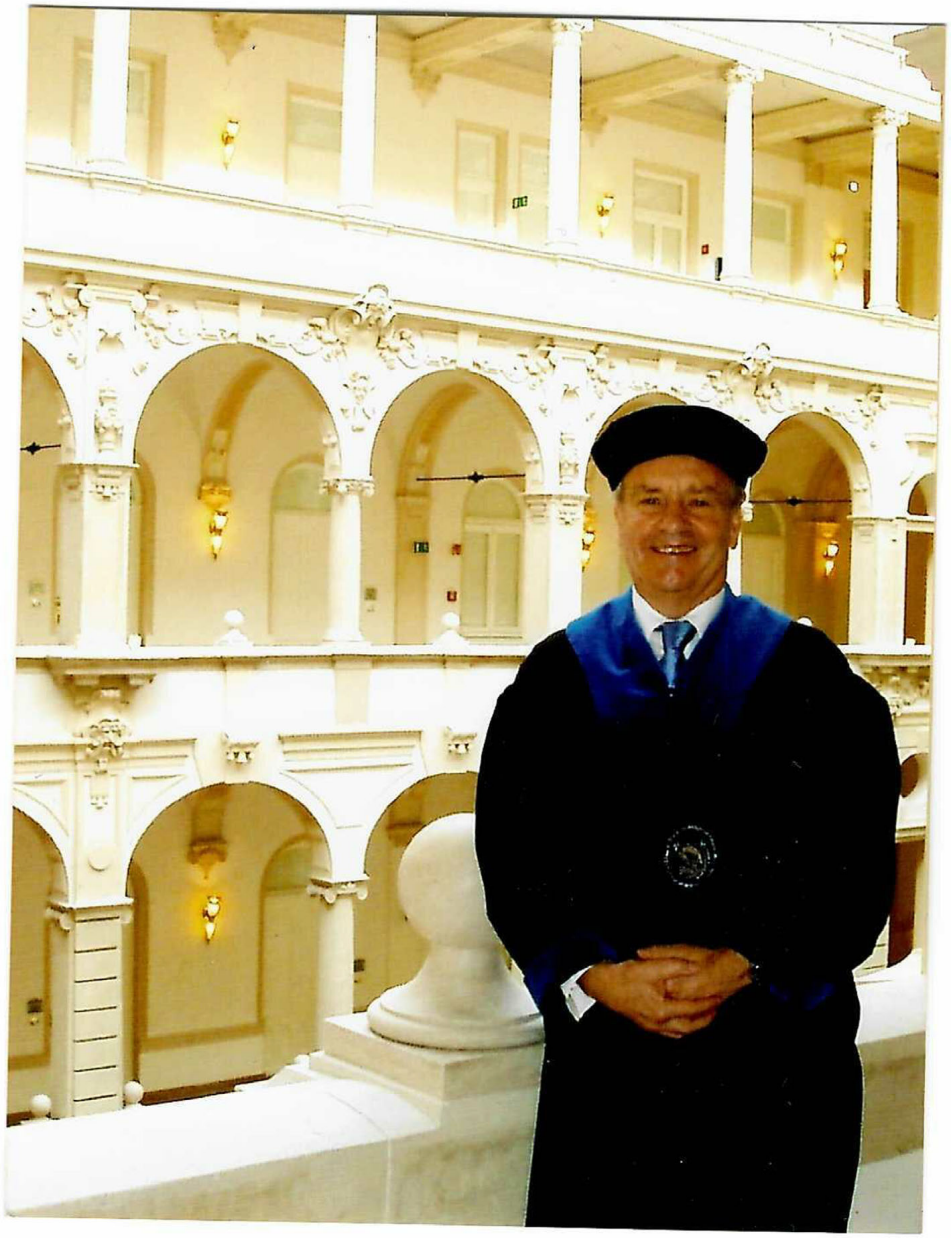
Rolf C. Gaillard is awarded *Doctor Honoris Causa* by Semmelweis University in Budapest, Hungary, in November 2006.

Among his many honours, Gaillard was awarded the title of *Doctor Honoris Causa* by Semmelweis University in Budapest, Hungary, in November 2006 (Figure 2). Rolf has acted for many years as a distinguished visiting endocrinologist at the Argentinian Endocrinology and Metabolism Society.

## 7 CONCLUSION

Rolf Christian Gaillard (1944–2011) not only was a clinician, a scientist, and an academic leader whose career contributed significantly to the development of modern European endocrinology and neuroendocrinology, but is also remembered as a brilliant and very pleasant colleague.

## AUTHOR CONTRIBUTIONS

**Eduardo Spinedi:** Conceptualization; writing – original draft. **François Pralong:** Conceptualization; writing – original draft. **Philippe Chanson:** Conceptualization; writing – original draft; writing – review and editing.

## CONFLICT OF INTEREST STATEMENT

The authors declare no conflicts of interest.

## Data Availability

Data sharing not applicable to this article as no datasets were generated or analysed during the current study.
